# Peripheral Heart Blocks Associated with Myocardial Infarcts: Clinical Diagnosis Based on Experimental Findings

**DOI:** 10.2174/157340308784245784

**Published:** 2008-05

**Authors:** Gustavo A Medrano, Alfredo de Micheli, Pedro Iturralde

**Affiliations:** Instituto Nacional de Cardiología “Ignacio Chávez”, Juan Badiano 1. 14080. México, D. F.

**Keywords:** Peripheral monofascicular blocks, Peripheral polyfascicular blocks, Ventricular depolarization in monofascicular blocks, Ventricular depolarization in left bifascicular blocks, Ventricular depolarization in trifascicular blocks, Left bifascicular blocks associated with dead myocardium, Trifascicular blocks associated with dead myocardium.

## Abstract

Septal necrosis + peripheral left blocks. Because of an extensive septal necrosis, the manifestation of the initial ventricular activation forces decreases in the precordial leads. With left bifascicular block (LASB + LPSB), the first ventricular activation forces become more evident and the electrical signs of septal necrosis can be concealed. In the presence of a trifascicular block, manifestation of the first ventricular electromotive forces diminishes again and the electrical signs of septal necrosis become evident once more. Small Q waves are present in leads V_1 _to V_4_.

Extensive anterior necrosis + peripheral blocks. This necrosis is manifested by QS complexes from V_2_ to V_6_. An associated left bifascicular block reduces the electrical manifestation of dead tissue: QS complexes persist only in V_3_ and V_4_. In turn, a coexisting trifascicular block causes the presence of QS complexes from V_2_ to V_5_.

Posteroinferior necrosis + peripheral blocks. Electromotive forces of the ventricular activation shift upward, due to a posteroinferior necrosis and QS or QR complexes are recorded in leads aVF, II and III. An associated left bifascicular block displaces the main electromotive forces downward, posteriorly and to the left, due to a delay of the posteroinferior activation fronts. The ventricular complexes become positive and wider in all leads, reflecting the potential variations of the inferior portions of the left ventricle: aVF, II, III, sometimes V_5_ and V_6_. Consequently, the electrical signs of necrosis are reduced or abolished. Due to a trifascicular block, wide and slurred QS complexes are recorded in aVF, II, III and sometimes in V_5_ and V_6_.

## INTRODUCTION

Fascicular or peripheral blocks can be diagnosed, based on two types of data: morphologically by the slurred vertex of the R wave only in leads exploring the affected regions and chronologically by the prolonged time of onset of the intrinsicoid deflection (TOID) in these leads. It seems correct to define as bifascicular block the one concerning two subdivisions of the same bundle, right or left, and as bilateral block that involves the right and left conduction systems.

It is justified to diagnose ventricular peripheral blocks in clinical tracings on the basis of experimental findings. For this, we produced a left anterior subdivision block (LASB) [1-4], as well as a posterior one (LPSB) [5,6], in dog hearts. Other authors have studied the ventricular depolarization process, in the presence of left peripheral blocks, in humans [7]. We investigated also the bases for diagnosing right peripheral blocks experimentally [8] and clinically [9,10].

### Examples

LASB delays and modifies the ventricular depolarization process in the superior regions of the left ventricle. Consequently, the left basal vector III points upward and to the left. In fact, the ventricular activation process progresses from the middle left septal mass toward the upper left septal mass and the basal parietal wall Fig. (**1**). The resultant vector of this depolarization process is manifested directly only in aVL Fig. (**2**).

In turn, the right anterior subdivision block (RASB) affects the superoanterior region of the ipsilateral ventricle. The late depolarization of this ventricle occurs in the upper right septal mass and parietal wall, as well as in the ventricular outflow tract. Hence, the right III vector, directed upward and to the right, accounts for the S_I_, S_II_, S_III_ morphologies and is directly manifested in aVR and unipolar right thoracic leads, V_4R_ - V_6R_. Fig. (**3**) reproduces intra-cavitary tracings showing the delayed activated myocardium in the right ventricle (V) near the outflow tract (ablation catheter).

LPSB produces a delay of the activation process in the left posteroinferior septal mass and left diaphragmatic parietal wall. For this, the main resultant vector of the left ventricular depolarization (III left vector) is oriented downward and to the left. This vector is the last of the ventricular activation process and is seen in the lower left leads, i.e., aVF Fig. (**4**).

RPSB delays the activation of the posteroinferior right septal mass and of the adjacent right ventricular wall. It is manifested in leads V_3R _- V_6R_, MD (unipolar in right hypochondrium), ME (unipolar in epigastrium), and aVF in horizontal hearts.

## LEFT BIFASCICULAR BLOCK (LASB + LPSB)

In the presence of an uncomplicated left bifascicular block, the ventricular activation process advances forward [11,12]. Consequently, the activation delay is detected in leads I and aVL, as well as in leads III, aVF, and V_6_: TOID is prolonged in these leads. The segmental activation delays in the free left ventricular wall can be represented by a mean resultant vector.

On the left side of Fig. (**5**), three main resultant vectors of normal ventricular depolarization in the human heart, are drawn. On the right, the main resultant vectors of ventricular activation, in the presence of left bifascicular block [13], are represented. The first septal vector (1) is directed more upward and forward than under normal conditions. The second vector (2) is oriented to the left, forward and downward. This is produced by normal activation forces of the lower anterior regions of the free left ventricular wall and is manifested at 0.036 sec, i.e., earlier than the normal left second vector (0.040 sec). The third vector, the strongest, results from the sum of left basal and delayed posterior activation fronts. Manifestation of this vector is at 0.06 sec or later in the human heart. It points to the left, backward, and upward or downward depending on the predominance of the left anterior (3’) or posterior (3’’) subdivision block. The latter often predominates when a posteroinferior myocardial infarction is also present. The sequence and spatial orientation of the three main left vectors, described above, satisfactorily explain the electrocardiographic and vectorcardiographic curves. This fact is due to slow conduction (muscle-muscle) and irregularly distributed muscle fibers or to the stunned strands within conduction pathways in the ischemic myocardial area.

### LBFB + Septal Inactive Myocardium

When the septal inactive myocardium is associated with a left bifascicular block (LBFB), the activation process is delayed in the posterior and the anterosuperior portions of the interventricular septum [14]. Therefore, the manifestations of electromotive forces, originating in the undamaged myocardium at the posteroinferior two thirds of the interventricular septum, are increased. These electromotive forces are directed to the right and downward. The electromotive forces originating in the trabecular zone of the right ventricle, which is depolarized at about 20 msec [15], probably join these forces. This fact produces changes in the morphology of ventricular complexes, which become rS in the right precordial leads and in V_3_. However, the voltage of small R waves is reduced in V_2_ and V_3,_ and qRs morphologies are registered in V_4_, whereas the ventricular complexes are Rs or qR in V_5_ and V_6_. These changes could suggest the coexistence of a LBBB of intermediate or advanced degree; although, primary-type repolarization waves (Twaves) persist.

### LBFB + Extensive Anterior Inactive Myocardium

LBFB causes the inscription of small positive initial deflections in V_2_ and qR morphologies, with slurred R waves, in V_5_ and V_6_, whereas QS morphologies persist in V_3_ and V_4 _[16]. Hence the inactive myocardium seems less extensive than it actually is. In fact, the electromotive forces directed forward are increased, probably due to a greater manifestation of those originating in undamaged regions of the mid third of the interventricular septum and in the trabecular zone of the right ventricle. Moreover, the electromotive forces originating in the left anterosuperior septal mass develop between 30 and 40 msec in the dog heart because of LASB [1-4] and contribute to increase the voltage of R waves in the transitional leads. The increment of electromotive forces in the free left ventricular wall, which last longer due to ipsilateral bifascicular block, increases the voltage of R waves in the left precordial leads. Furthermore, a clear predominance of the electromotive forces originated in the posterior regions of both ventricles is established.

### LBFB + Posteroinferior Inactive Myocardium

Because of this association, the delay in the activation of the free left ventricular wall due to both left peripheral blocks, can balance again the electromotive forces of the ventricular depolarization [17]. Nevertheless, this activation is delayed. The resultant vector again points downward, to the left and backward, producing slurred R waves with prolonged TOID in V_6_. In this case, the ECG is similar to that of a LBBB of intermediate degree [18]. The first septal vector is manifested because important electromotive forces are produced in the mid third of the interventricular septum. These electromotive forces can be registered on the thoracic surface. The corresponding vector is oriented upward, forward, and to the right, as reflected by the presence of small Q waves in the standard leads, V_5_ and/or V_6_. Summarizing, the ventricular complexes become positive, wide, in the low leads and in the left precordial leads. Because of this, the electrocardiographic signs of necrosis are reduced or can disappear. The electrical curves could suggest the presence of an aberrant LBBB of intermediate degree, but the left intraventricular QS complexes allow us to discard this possibility.

#### Clinical Example

The ECG of Fig. (**6**), recorded in a 63-year-old man, is suggestive of left bifascicular block: R waves appear slurred in aVL and aVF, but the TOID is more prolonged in aVF (100 msec) than in aVL (55 msec). Moreover, the manifestation of the first septal vector persists in V_1_ and V_6_, while the morphology of the left intraventricular complexes (LVM) is normal: QS. In turn, the small slurred Q waves in aVF, lasting 40 msec, suggest the presence of inactive myocardium in the diaphragmatic left ventricular wall.

## TRIFASCICULAR BLOCK

This block is really a bilateral block, but is called “Trifascicular” following common usage (Usus pater, said the poet Horace). 

Under experimental conditions, it is possible to produce a trifascicular block, i.e., left bifascicular block + right bundle branch block, without an atrioventricular block. In fact, the excitation wave can arrive at the middle left septal mass through intermediate subdivisions, arising from the left main bundle. The delayed activation process in both ventricles reduces their asynchronism. For this reason, the unipolar morphologies in leads V_1_ and V_2_ resemble those of an intermediate degree right bundle branch block (RBBB) [19]. Otherwise, the chronological and morphological characteristics of the left bifascicular block are modified. In the presence of a trifascicular block, an opposition occurs between the electromotive forces originated in the free left ventricular wall, oriented to the left, and those engendered by the “jumping” of the depolarization fronts within the septum, directed to the right [20], and by the reversed activation of the right inferior septal mass because of the RBBB. In other words, there is a marked opposition between the third vector of RBBB and the third vector of the left bifascicular block. This opposition of electromotive forces is responsible for shifting the 0.06-sec vector forward and to the left. This fact is manifested by tall, slurred, and delayed R waves in all precordial leads. On the other hand, this gives rise to significant changes in the horizontal vectorcardiographic curve (VCG_H_). A prominent clockwise R_H _loop exists, with a delayed vertex crossing the Z axis anteriorly to the E point and at around 0.07 sec.

Manifestation of the first vector is delayed. It is oriented more to the right than in the presence of isolated RBBB. The left second vector is displaced forward. The fourth vector of RBBB still points forward and slightly to the right, which explains the presence of small S waves in V_6_, as well as R’ in V_1_ and, occasionally, late “r” waves in aVR. The direction of the fourth vector accounts also for the small S loop of the vectorcardiographic ventricular curves.

The main forces of the repolarization process are directed downward and backward. Nevertheless, T waves may change according to the underlying heart pathology.

Fig. (**7**) shows an outline of the main resultant vectors of the ventricular depolarization process in the presence of a trifascicular block. When a RBBB is associated with a LBFB, the first vector is slightly delayed and directed more to the right, the second vector shifts forward, the third vector of RBBB is counterbalanced by the main vector of the left bifascicular block. Therefore, the 0.06-sec vector of ventricular activation points to the left and forward and the fourth vector remains practically unchanged.

The most characteristic chronological and morphological features of vectorcardiographic ventricular curves, in the presence of a trifascicular block, are evident in the horizontal plane. The shift of the 0.06-sec vector forward and to the left causes the presence of high, slurred, and delayed R waves in all precordial leads. Slowness of the depolarization process in both ventricles reduces the asynchronism of their activation. Because of this, the ventricular morphologies in V_1_ and V_2_ are similar to those of intermediate degree RBBB [21-23]. On their side, the chronological and morphological characteristics of the left bifascicular block do not change significantly.

### Trifascicular Block + Septal Inactive Myocardium

When a trifascicular block coexists with septal inactive myocardium, the initial forces of ventricular depolarization, oriented forward and to the right, are reduced [24]. This change is due to a delay in the manifestation of the electromotive forces engendered in the right septal mass and in the trabecular zone of the right ventricle, because of RBBB. Consequently, the voltage of the initial R waves diminishes in the right precordial leads. Slurred and wide Q waves frequently appear in the transitional leads V_3_ and V_4_ or from V_1_ until V_4_. The presence of abnormal Q waves in these leads permits to diagnose the coexistence of septal inactive myocardium associated with a trifascicular block. Nevertheless, some authors have found Q waves in leads V_1_ until V_4_ and electrocardiographic signs of trifascicular block, apparently not associated with necrosis of the interventricular septum [25]. In these cases, areas of septal fibrosis Fig. (**8**) or enlargement of the right atrium could exist, responsible for the inscription of Q waves. In any case, high precordial unipolar recordings must be obtained in the third and fourth left intercostal spaces in order to discard the presence of pathological Q waves.

Electrocardiographic signs of septal necrosis become more evident in the presence of a trifascicular block than with a left bifascicular block. The behavior of intermediate forces of ventricular depolarization allows detecting the trifascicular block. These forces are oriented forward, as in the case of RBBB, and to the left, as in the case of LBBB. The changes in the fourth vector depend on the extent of septal inactive myocardium. This vector is now located in a middle position or distorted to the left, explaining the smaller voltage of the late R wave in the right precordial leads and V_3R_, just as can be seen in the presence of RBBB complicating a septal necrosis [26]. The signs of modified primary type ventricular repolarization persist.

If the septal inactive myocardium is very extensive, the mean vector of ventricular depolarization is directed now upward and forward, but it is always oriented to the left. Consequently, negative or predominantly negative ventricular complexes are registered in precordial leads: QS from V_2_ until V_5_ and qRS in V_6_, sometimes qR from V_1_ until V_6_. QS complexes in V_5_ and V_6_ could be due to local electrical effects picked up only in the near unipolar leads [27]. The complexes can be understood only if one considers that left precordial leads record the potential variations of damaged myocardial regions. Terminal forces of the ventricular depolarization are originated in the basal regions of the right ventricle, which are partially superimposed on the late forces developing in the superior third of the left ventricle [28]. The resultant vector of these forces is oriented upward and situated in a mid position. The ventricular electromotive forces adapt also thanks to the effect of the injured zone on the anteroinferior half of the interventricular septum, which can be represented by a local vector oriented downward, forward, and slightly to the left. On the other hand, an initial loop of butterfly wings type, lasting around 35 msec, is inscribed in the horizontal vectorcardiographic curve.

### Trifascicular Block + Posteroinferior Inactive Myocardium

In these conditions, the ventricular depolarization can be synthesized in the following points [17]:

1. The first septal vector, of increased voltage, is oriented upward, forward, and to the right. This vector is responsible for the initial negativity of ventricular complexes in the standard leads and aVF.
2. The delayed second vector, originated in the free left ventricular wall, is directed upward, backward, and to the left (around -80^o^ in both frontal and horizontal planes).
3. The third vector represents the slow and delayed depolarization of the interventricular septum. It is oriented upward and to the right, at times backward, at times forward. In the human heart, this vector points less upward than in the dog heart.
4. The fourth vector, originated by the depolarization of basal regions of the free ventricular walls and of high portions of the interventricular septum, is directed to the right, forward and slightly upward.

Therefore, the ventricular complexes are slurred, and wide QS are present in L_II_, L_III_, and aVF (signs of posteroinferior transmural necrosis). Sometimes, these complexes are recorded in V_5_ and V_6_ too, mimicking a lateral inactive myocardium. Delay and slowness of the ventricular depolarization, due to these associated blocks, are manifested through slurred R waves and unequally prolonged TOID in leads exploring damaged myocardial regions. The orientation of the main resulting vectors of ventricular depolarization, in the presence of a trifascicular block, is shown in Fig. (**7**).

### Clinical Example

The ECG of Fig. (**8**) was recorded in a 36-year-old man with cardiomyopathy. The inferior tracing shows a trifascicular block. Sinus rhythm persists with a slight increase of P-R from 0.16 sec to 0.18 sec and a QRS-duration of 0.15 sec. ÂQRS_F_ is at -20^o^. Slurred qR complexes are registered in leads I and aVL, where the onset of the intrinsicoid deflection is around 0.08 sec, rS morphologies are inscribed in leads III and aVF. The TOID is around 0.04 sec in leads III and aVF. RBBB is recognizable in lead V_1_. The unipolar morphologies are rs in aVR, qR in V_5_ and V_6_ with TOID= 0.06 sec. The diagnosis of trifascicular block is supported also by the orientation of ÂQRS_F_, the slurred R waves in leads aVF and V_6_, and the absence of a terminal R wave in aVR, which must be prominent in the presence of LASB associated with RBBB. SÂT is oriented downward, backward and slightly to the right, whereas in the previous ECG it was directed upward, forward, and to the left. The slurred Q waves, of 0.04 sec, in V_4_ and V_5_ suggest the presence of inactive septal myocardium.

The early diagnosis of a trifascicular block seems to be particularly useful due to an impending severe A-V conduction disturbance.

## COMMENT

It is very useful to establish the diagnosis of peripheral blocks, in clinical electrocardiograms, on the bases of experimental findings. Some years ago, we published the electrocardiographic manifestations of dead myocardium associated with peripheral monofascicular blocks, emphasizing that left ones, LASB and LPSB, can conceal the electrocardiographic signs of the inactive anteroseptal and posteroinferior myocardium, respectively [29].

We produced also left bifascicular and trifascicular blocks in canine hearts [11] and published data on the uncomplicated and complicated polyfascicular blocks [27]. When a left bifascicular block is present, the activation process is unequally delayed in the high lateral regions and in the posterior ones of the free left ventricular wall. Nevertheless, this process begins at the normal time in the middle left septal surface *via* intermediate strands of the left bundle. Consequently, a left bifascicular block, resembling an intermediate degree LBBB [18], can be detected by the unequally delayed time of onset of the intrinsicoid deflection in aVL, aVF, and V_6_, often by the manifestation of the first septal vector also. The vectorcardiographic ventricular curves are diphasic and show initial and terminal slurs. A left bifascicular block associated with an extensive anterior necrosis reduces the electrical manifestation of dead myocardium: QS complexes persist only in V_3_ and V_4_. This block, complicating a posteroinferior necrosis, causes the ventricular complexes to become positive and wider in all leads [27]. This fact is due to the potential variations on the inferior regions of the left ventricle: aVF, II, and III, sometimes V_5_ and V_6_. Therefore, the electrical signs of necrosis are reduced or concealed.

A trifascicular block delays the activation sequence in both ventricles. Therefore, the asynchronism between the electromotive forces of the ventricles is lessened [30]. This conduction disturbance can be diagnosed by electrocardiographic features, suggesting an intermediate degree RBBB [31,32], as well as a left bifascicular block [33]. If a trifascicular block coexists with an extensive anterior necrosis, it causes the inscription of QS complexes from V_2_ to V_5_. Wide and slurred QS complexes are recorded in aVF, II, III, and sometimes also in V_5_ and V_6 _when the mentioned conduction disturbance is associated with a posteroinferior necrosis.

## Figures and Tables

**Fig. (1):**
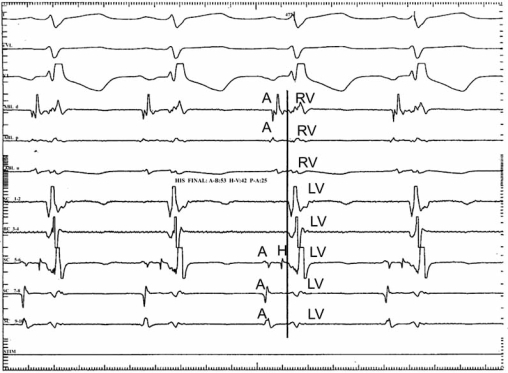
LASB. Intracavitary tracings show the delay of the ventricular depolarization process in the superior regions of the left ventricle (the last trace). A= Atrium. H= His. LH = Left His. RV= Right ventricle. LV = Left ventricle.

**Fig. (2):**
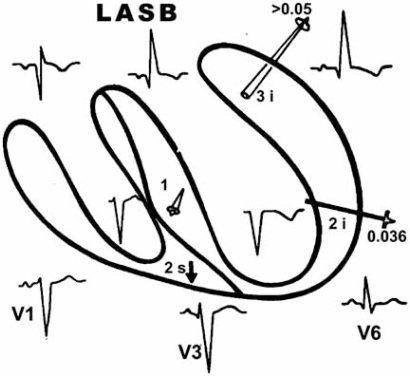
LASB. The resultant vector of left ventricular depolarization (3i) is oriented upward and to the left.

**Fig. (3):**
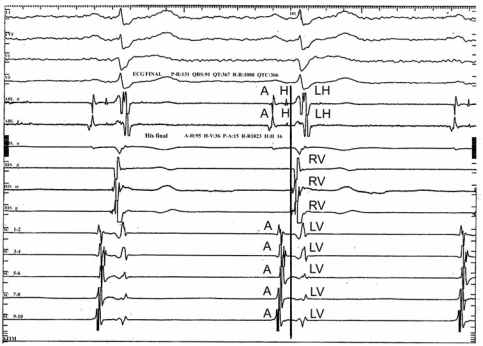
RASB. Intracavitary tracings showing the most delayed activation zone in the outflow tract of the right ventricle (fifth trace). A= Atrium. LV = Left ventricle. RV = Right ventricle. LH= Left His.

**Fig. (4):**
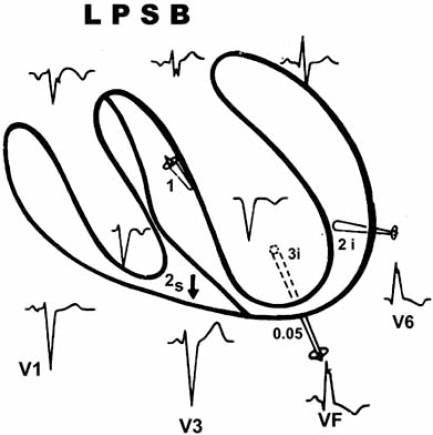
LPSB. Intracavitary tracings show a delay of the ventricular activation process occurring in the posteroinferior left septal mass and in the diaphragmatic wall of the left ventricle. The main resultant vector (3i vector) is oriented downward and to the left.

**Fig. (5):**
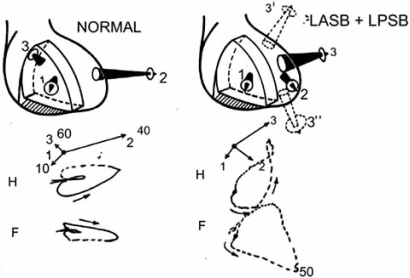
The main resultant vectors of the ventricular activation process in the presence of a left bifascicular block.

**Fig. (6):**
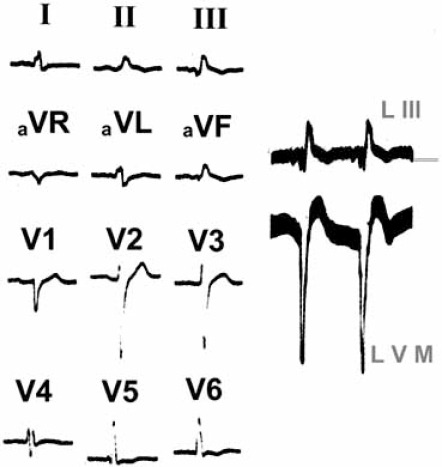
ECG registered in a 63-year-old man, suggesting a left bifascicular block. The morphology of left intraventricular complexes (LVM) is normal: QS.

**Fig. (7):**
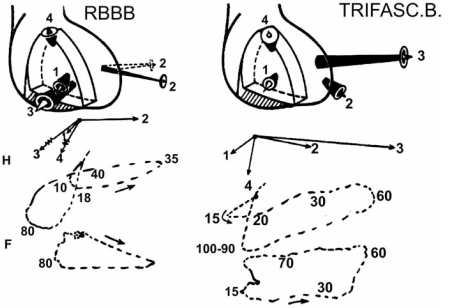
The main resultant vectors of ventricular depolarization in the presence of a trifascicular block. The time of manifestation of the resultant vectors of ventricular depolarization are expressed in msec.

**Fig. (8):**
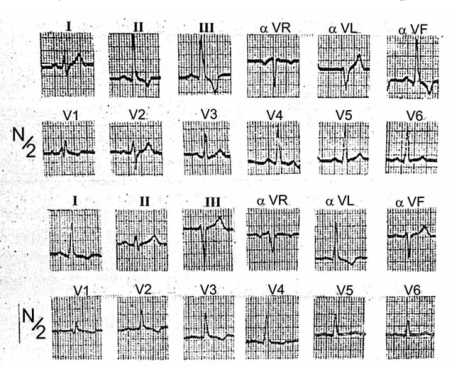
ECG corresponding to a 36-year-old man with cardiomyopathy and trifascicular block.

## ABBREVIATIONS

TOID: = Time of onset of intrinsicoid deflection
LASB: = Left anterior subdivision block
LPSB: = Left posterior subdivision block
LBFB: = Left bifascicular block
LBBB: = Left bundle branch block
RBBB: = Right bundle branch block
LVM: = Left intraventricular morphology
VCG_H_: = Vectorcardiogram in the horizontal plane
VCG_F_: = Vectorcardiogram in the frontal plane
